# Energy Modulated Photon Radiotherapy: A Monte Carlo Feasibility Study

**DOI:** 10.1155/2016/7319843

**Published:** 2016-02-09

**Authors:** Ying Zhang, Yuanming Feng, Xin Ming, Jun Deng

**Affiliations:** ^1^Department of Biomedical Engineering, Tianjin University, Tianjin 300072, China; ^2^Department of Therapeutic Radiology, Yale University, New Haven, CT 06511, USA

## Abstract

A novel treatment modality termed energy modulated photon radiotherapy (EMXRT) was investigated. The first step of EMXRT was to determine beam energy for each gantry angle/anatomy configuration from a pool of photon energy beams (2 to 10 MV) with a newly developed energy selector. An inverse planning system using gradient search algorithm was then employed to optimize photon beam intensity of various beam energies based on presimulated Monte Carlo pencil beam dose distributions in patient anatomy. Finally, 3D dose distributions in six patients of different tumor sites were simulated with Monte Carlo method and compared between EMXRT plans and clinical IMRT plans. Compared to current IMRT technique, the proposed EMXRT method could offer a better paradigm for the radiotherapy of lung cancers and pediatric brain tumors in terms of normal tissue sparing and integral dose. For prostate, head and neck, spine, and thyroid lesions, the EMXRT plans were generally comparable to the IMRT plans. Our feasibility study indicated that lower energy (<6 MV) photon beams could be considered in modern radiotherapy treatment planning to achieve a more personalized care for individual patient with dosimetric gains.

## 1. Introduction

High energy mega-voltage photon beams (10 MV and higher) are suitable to treat deep-seated tumors due to their penetration power and skin sparing. However, the large exit dose outside the target volume and the neutron contamination can be of great concerns in clinical practice. In addition, high energy photons have a pronounced dose build-up or build-down effect at the heterogeneous medium interface due to the increased range of secondary electrons, causing increase of beam penumbra, overdose of normal tissues, and underdose of target edge volumes [1–3]. On the other hand, low energy (6 MV and lower) photon beams have narrow penumbra which are suitable to deliver a tight dose distribution around the target. Low exit dose also benefits the critical structures adjacent to the target.

In recent years, there has been increasing interest in exploring the potential benefits of lower energy photon beams, such as Cobalt-60 gamma ray (energy peaks at 1.17 and 1.33 MeV) and lower energy (<6 MV) X-rays. In 2007, Keller et al. showed that intermediate energy X-rays (1.2 MV) combined with small fields can reduce the radiological penumbra in intracranial stereotactic radiosurgery (SRS), which could be substantially beneficial for improved dose distribution homogeneity and better sparing of critical structures [4]. Fox et al. have compared Cobalt-60 gamma ray with 6/18 MV photons and demonstrated that nearly identical intensity-modulated radiotherapy (IMRT) plans can be achieved between Co-60 and 6 MV photons [5]. Dhanesar et al. have indicated that Co-60 Tomotherapy is capable of providing the state-of-the-art conformal dose delivery in both phantom and clinical planning studies [6]. If the build-up effect is of concern when the target volume is near air cavities, Behrens suggested that 4 MV should be preferred over both 6 MV and 8 MV [7]. More recently, Dong et al. investigated a 2 MV FFF beam for extracranial robotic IMRT. Their results demonstrated that the dual energy plan (2 and 6 MV) had the best dosimetry in terms of equivalent target coverage and improved organs-at-risk (OARs) sparing, followed by 2 MV only and 6 MV only plans [8]. In addition, Zhang et al. studied the effects of 3 MV photon beams for lung cancer treatment [9]. By comparing 6 MV, 3 MV, and dual energy plans for 31 lung cancer patients, they concluded that 3 MV photon beams have statistically significant dosimetric benefits in terms of improved tumor coverage and reduced doses to the adjacent critical structures [9].

The potential of mixing photon beam energy has also been explored in some early investigations. Malhotra et al. showed that, for the prostate cases, the mixed energy photons of 6, 10, 15, or 18 MV would be better in terms of integral dose than using either low or high energy photons alone [10]. In a study by St-Hilaire et al., beam energy was added as an optimization parameter in an automatic aperture-based inverse planning system. Their work demonstrated that energy optimization could produce plans of better quality with less peripheral dose and fewer monitor units (MUs) for prostate and lung tumors [11]. Park et al. also showed the advantage of energy- and intensity-modulated radiotherapy in terms of less integral dose while maintaining the plan quality [12]. In their other study, a cylindrical energy modulator with adjustable thickness of mercury along the beam axis was used to replace the flattening filter and to modulate the photon beam energy. They further commissioned a virtual machine in a treatment planning system (TPS) based on Monte Carlo simulated photon beams going through the energy modulator of various thickness of mercury [13]. While this novel energy modulator provided a way to generate mean energy adjustable photon beams, the incorporation of this modulator with toxic mercury into a modern Linac treatment head would pose a series of engineering, manufacturing, and safety challenges.

Based on all the previous observations, we propose a new treatment modality termed energy modulated photon radiotherapy (EMXRT) to systematically explore the benefits of lower energy photon beams for cancer radiotherapy. In EMXRT, a broad range of lower energy photons (i.e., 2, 3, 4, 5, 6, 10 MV) were considered as variables during the inverse planning process, with the consideration of tumor size, effective path length inside the patient for a certain gantry angle, and skin dose as well as beam exit dose. Higher energy photons (>10 MV) were not chosen in this study to avoid the overlapping with previous investigations [10, 12]. All the energy beams were modeled using an EGS4/BEAM Monte Carlo code based on the specifications of an actual linear accelerator. An in-house inverse planning system based on gradient search algorithm has been developed and used to optimize photon beam intensity based on presimulated Monte Carlo pencil beam dose distributions in patient anatomy. The pros and cons of EMXRT have been discussed in six clinical cases with respect to the conventional IMRT treatments.

## 2. Material and Methods

### 2.1. Patient Selection and Workflow

**Figure 1:**
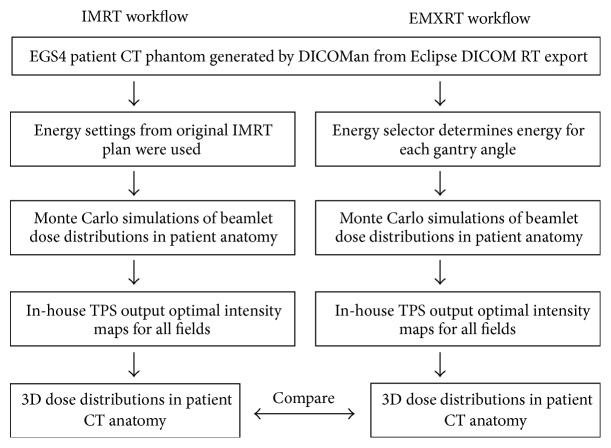
Comparison of IMRT workflow with EMXRT workflow in this study.

Six cancer patients treated with IMRT were retrospectively selected to represent a variety of tumors (i.e., prostate, brain, lung, spine, thyroid, and head and neck) and locations. As shown in Figure 1, a patient CT phantom was first created from planning CT images, with the target and other critical structures delineated in a commercial treatment planning system (TPS). For each patient, the original IMRT treatment was planned with a Varian Eclipse TPS v10.0.28 on a Varian Trilogy Linac (Varian Oncology Systems, Palo Alto, CA). Accordingly, an EMXRT treatment was planned with our in-house inverse planning system based on the presimulated Monte Carlo pencil beam dose distributions of a variety of lower energy photon beams. For the sake of comparison, we have also replanned the IMRT using the same in-house inverse planning system with the same beam energy set as in the original Eclipse plan. Once the inverse planning was done, Monte Carlo method was used to simulate the dose distributions in patient anatomy for both IMRT and EMXRT plans. Specifically, a benchmarked EGS4 user code, MCSIM, was used to simulate the 3D dose distributions in the obtained patient CT phantoms with all the beam configurations, leaf leakage, and transmission being considered [14, 15]. Finally, the two plans were evaluated and compared in terms of target coverage and normal tissue sparing.

### 2.2. Monte Carlo Simulation and Modeling

It is widely accepted that the Monte Carlo method is one of the most accurate dose calculation methods available for radiotherapy. In this study, an EGS4/BEAM code has been used to simulate the particles emanating from a Varian Linac treatment head. The machine configurations were the same as those of the clinical 6 MV photon beams; only the energy of incident electron beam was set to be the nominal energy of 2, 3, 4, and 5 MeV, respectively [16, 17]. The configuration of 10 MV was different as the flattening filter for 10 MV was used. The phase space data was first scored above the photon jaws located at 28 cm downstream from the target and then EGS4/BEAMDP was used to derive the multiple source model for all the energy beams. The obtained multiple source models consisted of detailed numerical description of the energy spectrum, spatial distribution, fluence distribution, source location, shape, and size of each source for a particular treatment head [18, 19]. The multiple source models have been shown equivalent to the phase space data in representing the photon beams from the Linac treatment head and replicating the dose distributions in water, yet eliminating the inconvenience of large data transfer and latent variance related to the phase space [18–20].

EGS4/MCSIM has been used for 3D dose calculations in both water phantoms and patient CT anatomy in this study. To demonstrate the beam characteristics of low energy photons, a series of percent depth dose curves (PDDs) and dose profiles along *x*-axis at various depths has been simulated with MCSIM in a rectilinear water phantom. In addition, the PDDs and dose profiles of 6 and 10 MV photon beams in water were measured and compared with Monte Carlo simulations. To simulate 3D dose distributions in patient anatomy, an in-house DICOM tool named DICOMan was used to construct a realistic CT phantom based on the planning CT images and contoured structures of individual patient exported from Eclipse TPS via DICOM RT protocol [21]. To enhance the efficiency of Monte Carlo simulations, a series of variance reduction techniques has been implemented in MCSIM such as photon interaction forcing, Russian roulette, particle splitting, and electron track repeating. In all Monte Carlo simulations, the energy cutoffs for electrons (ECUT) and photons (PCUT) and the energy thresholds for *δ*-ray production (AE) and bremsstrahlung production (AP) were set as ECUT = AE = 700 keV and PCUT = AP = 10 keV, respectively. A better than 1% statistical uncertainty (1*σ*) was achieved for beam PDDs, profiles, and the target voxels in dose simulations. The benchmark results of EGS4/MCSIM have been reported previously [14, 15].

### 2.3. Energy Modulated Photon Radiotherapy Treatment Planning

#### 2.3.1. Energy Selector

**Figure 2:**
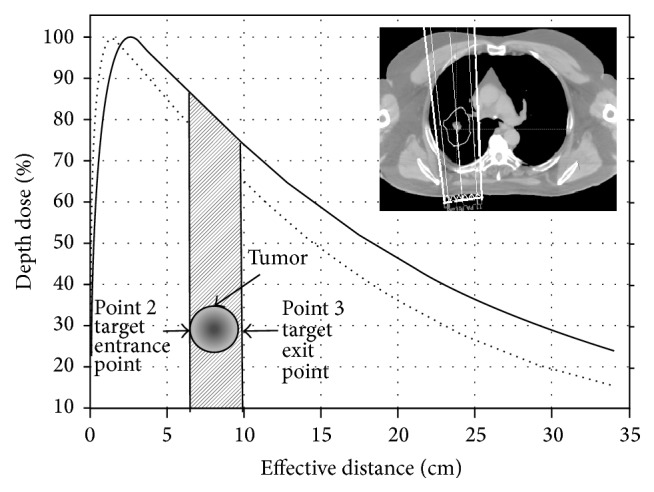
Diagram of energy selector correlation coefficient (CC) model. The PDD curves for high and low energy photon beams were shown with solid and dotted lines. A spherical tumor model was located and target entrance and exit points (points 2 and 3) were marked by the arrows. In a real patient, the beam energy was determined by looking up the energy table for a certain effective path length and tumor size (inset).

For EMXRT treatment planning, an energy selector was developed to provide a convenient way to determine an appropriate energy for photon beams at different gantry angles in the studied energy range. As shown in Figure 2, for any photon beam impinging on the patient contour from outside and transiting the tumor volume inside, there were four special points to be considered by the energy selector, that is, the entrance point to the contour (point 1, corresponding to skin dose), the entrance point to the target (point 2, target entrance dose) and exit point from the target (point 3, target exit dose), and finally the exit point from the contour (point 4, exit dose). The skin dose is one of the limiting factors during the treatment planning, especially when lower energies were considered. In addition, the effective path length (EP) and tumor size (TS) can be determined based on the distances between points 1, 2, and 3. The target dose homogeneity can be represented by the target entrance and exit doses. The exit dose at point 4 can be a general representative of the doses to the critical structures and healthy tissues downstream.

Ideally, a uniform dose distribution in the target with minimum doses delivered to the OARs would be expected. However, when treatments are delivered to the patients, the radiation beam may intersect different mediums such as bone, muscle, fat, lung, and air cavities. Because of the inhomogeneity of human body, we used the effective path length (EP) in this study, which was obtained by normalizing the physical path length to a water-equivalent path length from patient skin to the isocenter. Tumor size (TS) was calculated as the water-equivalent length from the target entrance point to the target exit point. A wide range of tumor size from 2 to 16 cm was investigated in this study to accommodate most of the clinical cases.

A spherical tumor model merged in water, whose diameter was equal to tumor size (TS) and whose center was effective path length (EP) away from the entrance, was used to imitate various clinical situations where impinging beams intersected four points along the beam direction with the contour and the tumor. Correlation coefficients (CC), as defined in (1), were calculated between the ideal doses *D*_ideal_(*i*) and the realistic doses *d*_PDD_(*i*) of different energies for these four points. Particularly, for the ideal situation, *D*_ideal_(1) and *D*_ideal_(4) were set to be 10% while *D*_ideal_(2) = *D*_ideal_(3) = 100% for the ideal homogeneous dose distribution in the tumor. For the realistic situation, *d*_PDD_(*i*) were dose values at four points derived from PDD curves for a given beam energy, TS, and EP in water. Weighting factor *ω* was assigned to each point to show different importance when considering photon energy for a radiation treatment. According to our experience, we assigned 0.5 to skin dose but 1.0 to the other three points in this study. CC was calculated as below:

(1) $$\begin{array}{c} CC=\frac{\sum_{i=1}^{4}{\omega \left(i\right)\times D}_{ideal}\left(i\right)\times d_{PDD}\left(i\right)}{\sqrt{\sum_{i=1}^{4}{D_{ideal}\left(i\right)}^{2}}\times \sqrt{\sum_{i=1}^{4}{d_{PDD}\left(i\right)}^{2}}}. \end{array}$$

The energy corresponding to the maximum CC was chosen as the optimal energy for this EP/TS scenario. Following the same fashion, an energy look-up table has been generated for various EP/TS configurations, which can be readily used in energy selection for different patient cases. It should be pointed out that some common knowledge was also applied in the implementation of energy selector. For example, for some deep-seated tumors with very large EP (>18 cm), regardless of tumor size, high energy (10 MV) photon beam would be chosen. Moreover, EP should always be larger than the radius of tumor (1/2 of TS).

#### 2.3.2. Inverse Planning

With multiple source models as beam input, the dose deposition coefficients (DDCs) were scored to yield the dose distribution from each beamlet (1 cm × 1 cm) to each structure voxel in the patient for the specific beam energy at specific gantry angle. Therefore, for the dose to the voxel *i*, *d*_*i*_ is given by the summation of the DDCs from all beamlets weighted by their own weights as shown in

(2) $$\begin{array}{c} d_{i}=\alpha_{i}\cdot x=\overset{N_{b}}{\underset{j=1}{\sum }}a_{ij}x_{j}, \end{array}$$

where *N*_*b*_ is the total number of beamlets for all beams, *a*_*ij*_ represents the DDC from the *j*th beamlet to the *i*th voxel, and *x*_*j*_ is the weigh for the *j*th beamlet.

Then, the weighting factors for all beamlets were optimized via an in-house inverse planning code so that the desired dose distribution could be achieved. Further details of the optimization method used in this work can be found elsewhere [22]. The system utilizes a steepest descent search algorithm, with a quadratic objective function augmented by dose volume constraints of both target and OARs, as shown below:

(3) Fobjx,r=fobjtx+Ptx+Pcx=∑i=1Ntdi−p0t2+r∑k=12ωkt∑i=1Ntδidi−pkt2+r∑n=1Mωnc∑k=1Lωn,kc∑i=1Nncδidi−pn,kc2,

where *p*_0_^(*t*)^, *p*_*k*_^(*t*)^, and *p*_*n*,*k*_^(*c*)^ are the prescribed dose to the target, the *k*th dose volume constraints for the target, and the *k*th dose volume constraints for the *n*th critical structure. *N*(*t*) and *N*_*n*_^(*c*)^ are the total number of dose points inside the target and the *n*th critical structure, respectively. *ω*_*k*_^(*t*)^, *ω*_*n*_^(*c*)^, and *ω*_*n*,*k*_^(*c*)^ are the weights assigned to the *k*th dose volume constraints for target, the *n*th critical structure, and the *k*th dose volume constraint for the *n*th critical structure, respectively. *δ*_*i*_ is a flag, defined as 1 when the constraint is violated and 0 when it is not. *M* is the number of critical structures considered in the optimization and *L* is the number of dose volume constraints for the *n*th critical structure. The factor *r* will be increased as the iterations proceed. Thus, the penalty assigned to the constraint violations becomes increasingly severe as represented by *r*. In each iteration, the weights of beamlets were adjusted along the negative of the gradient to minimize the objective function. The optimization will stop once the value of the objective function keeps almost unchanged for several rounds or the predefined time limit is violated.

To simplify the optimization, the complete irradiated area outlines (CIAO) extracted from the IMRT plan in Eclipse, which were the general beam shape opened for fitting the target, were used as the initial guess such that the intensity of the beamlets inside the corresponding CIAO was set to be one while the outside was set to be zero.

### 2.4. Plan Evaluation

Dose volumetric analysis was performed to compare the new modality EMXRT with the conventional IMRT. To start with, the dose volume histograms (DVHs) of both IMRT and EMXRT plans were normalized such that 100% isodose lines would encompass 95% of target volume. For PTVs, the mean dose (*D*_mean_) and the dose levels covering 2% and 98% of the PTV volume (*D*_2%_ and *D*_98%_) were calculated and compared. In addition, the homogeneity index (HI), defined as HI = (*D*_2%_ − *D*_98%_)/*D*_50%_, was used to evaluate the homogeneity of the PTV coverage. Some specifically selected dosimetric parameters of OARs were generated from DVHs and analyzed. The integral dose was also calculated for surrounding normal tissue by integrating the doses over all the voxels within the patient volume while excluding the PTV. Finally, the isodose distributions in the axial, coronal, and sagittal views were compared between the two modalities.

## 3. Results

### 3.1. Benchmark Results

**Figure 3:**
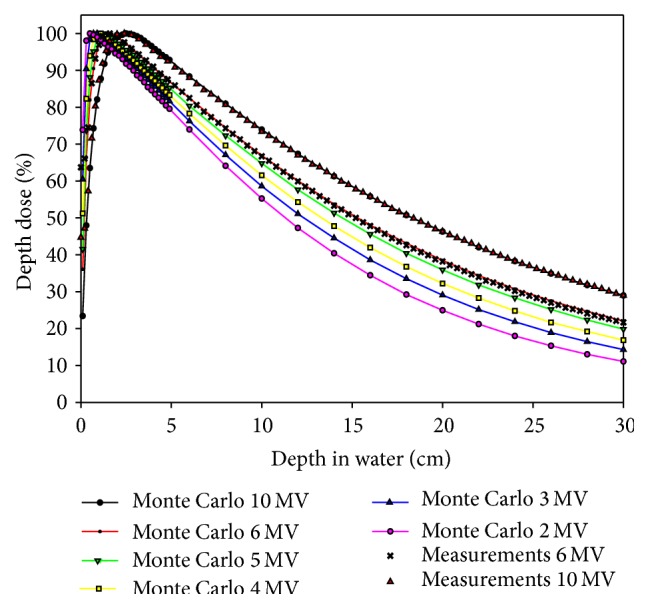
Comparison of percentage depth dose (PDD) curves of various beam energies in a water phantom between Monte Carlo simulations (lines with symbols) and ion chamber measurements (symbols) for the field of 10 × 10 cm^2^ at 100 cm SSD.

Figure 3 compared the percent depth doses of various beam energies in water between the Monte Carlo simulations (line with symbols) and the measurements (symbols) for the field of 10 cm × 10 cm at 100 cm source-to-surface distance (SSD). Note that all the Monte Carlo simulations were performed with MCSIM with multiple source models as beam input, and the depth dose measurements were carried out in an IBA blue phantom water scanning system and scanned with two CC-13 ionization chambers (IBA Dosimetry America, Bartlett, TN) with one as field detector and the other as reference detector. The agreement between the measurements and the Monte Carlo results for 6 and 10 MV have been validated in our previous work regarding multiple source modeling for accurate Monte Carlo dose calculations [20]. Part of the percentage depth dose data and the surface dose for all the beams were listed in Tables 1 and 2, respectively. As beam energy decreased, the TPR_20,10_ decreased from 0.627 for 10 MV to 0.446 for 2 MV. Although lower energy photons carried less penetrating power as indicated by the smaller TPR_20,10_, it could be advantageous in some situations where critical structures were to be spared in the downstream.

**Figure 4:**
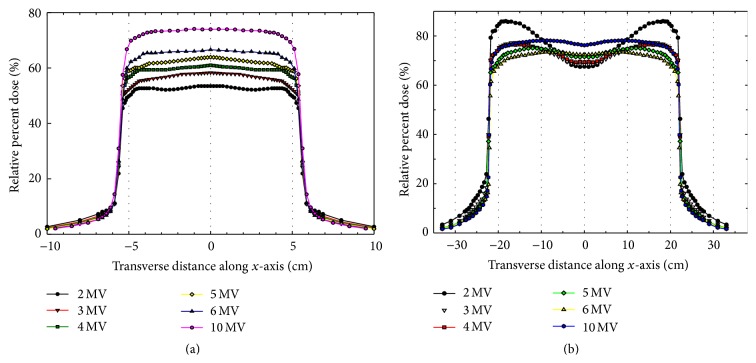
Comparison of Monte Carlo simulated beam profiles at depth of 10 cm in water for photon beams of various energies at 100 cm SSD with the field size of (a) 10 × 10 cm^2^ and (b) 40 × 40 cm^2^.

Beam profiles for all the energies were also generated from Monte Carlo simulations at 10 cm depth for the 10 cm × 10 cm (Figure 4(a)) and 40 cm × 40 cm (Figure 4(b)) field sizes. For 10 cm × 10 cm open field, the overall flatness of the beam profiles was considered clinically acceptable, while, for the 40 cm × 40 cm open field, horn shaped profiles were observed for the lower energies, such as 2 MV, 3 MV, and 4 MV. This uneven flatness was primarily caused by the nonoptimized design of flattening filter for these energies used in Monte Carlo beam simulation as well as the inherent fluctuation in the particle fluence scored in the phase space for beam modeling. However, in this work, the uneven fluence will be handled by assigning the appropriate weighting factors for the beamlet dose distributions during the inverse planning process.

### 3.2. Energy Selector

In this work, the correlation coefficients (CC) were calculated for all the available beams for each scenario with EP ranging from 2 to 21 cm and TS from 2 to 16 cm. The energy with the maximum CC was chosen as the estimated energy for the specific EP/TS configuration. An energy look-up table shown in Table 3 was determined by the proposed energy selector. Overall, higher energy photons were preferred for large-sized tumors and/or deep-seated ones, consistent with conventional clinical practice. 2 MV was not indicated in this energy table due to its very high skin dose and steep PDD curve. 5 MV was preferred in a large number of scenarios when the skin dose, the exit dose, and the tumor dose homogeneity were considered. For tumors larger than 11 cm in diameter or located as deep as 18 cm, the suitable photon energy was 10 MV as determined by the energy selector, which is consistent with our expectation.

For all six cases, the EP and TS of all beam angles were measured in Eclipse and listed in Table 4. In IMRT plans, 10 MV photon beams were used for the prostate patient and 6 MV photon beams for all the other patients. In EMXRT plans, beam energies were determined according to the energy look-up table in Table 3. Compared with IMRT plans, more beams of lower energy and multienergy photon beams were employed in EMXRT plans. Specifically, in the prostate EMXRT plan, 10 MV was selected for the left and right posterior oblique beams while 5 MV was preferred for the anterior posterior and two anterior oblique beams. For the brain case, even the effective path lengths for all the beam angles were relatively small, 5 MV instead of lower energy photon beams were determined by the energy selector as the large volume of tumor would require higher energy photons with stronger penetration power. For the other four cases, as tumor sizes largely fell into the range of 4 to 8 cm, 5 MV photon beams were dominantly chosen by the energy selector based on the consideration of tumor dose coverage and sparing of critical structures adjacent to the tumor.

### 3.3. Plan Evaluation

**Figure 5:**
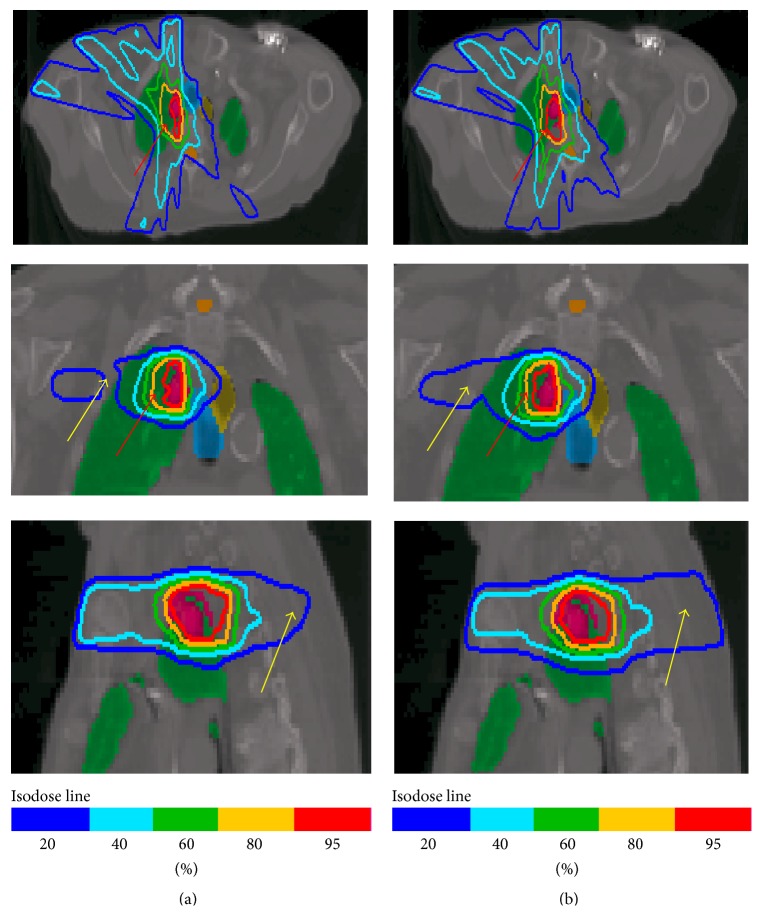
Comparison of dose distributions between EMXRT (a) and IMRT (b) plans for the lung cancer patient in the axial, coronal, and sagittal views through the center of the target region. The planning target volume (PTV), lung, and trachea were shown with pink, green, and blue regions, respectively. Notable differences of the dose distribution between the two plans were shown using red (95% isodose) and yellow arrows (20% isodose).

**Figure 6:**
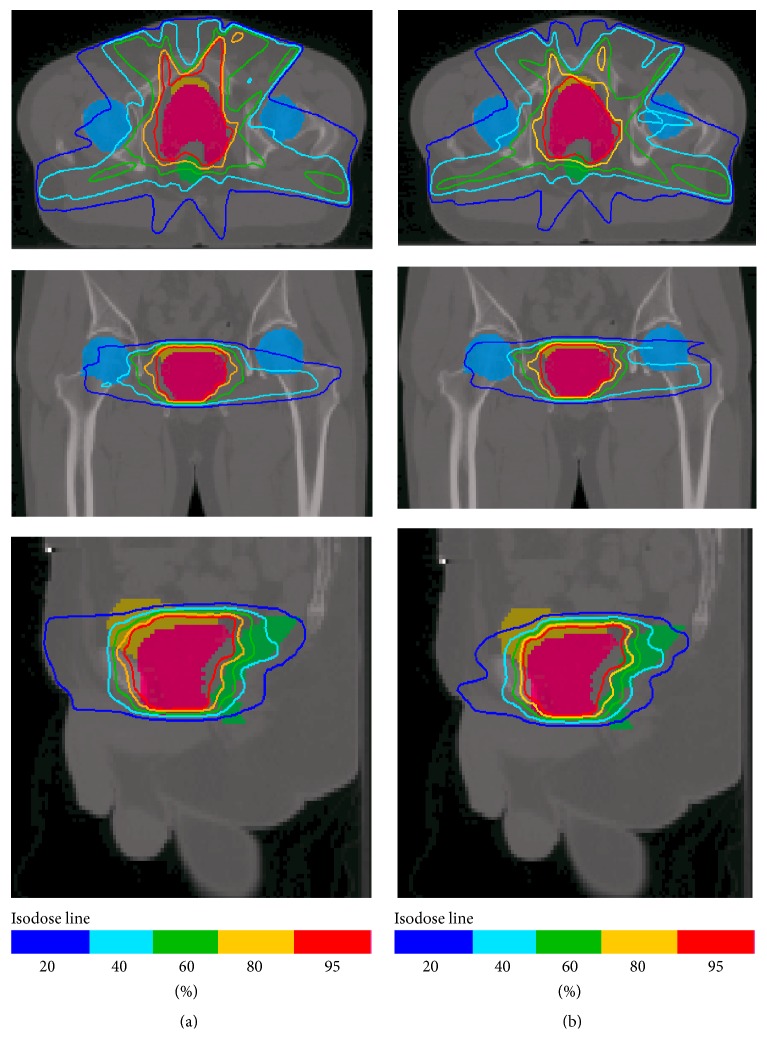
Comparison of dose distributions between EMXRT (a) and IMRT (b) plans for the prostate cancer patient in the axial, coronal, and sagittal views through the center of the target region. The PTV, rectum, bladder, and femur head were shown with pink, yellow, green, and blue regions, respectively.

The dose distributions along the axial, coronal, and sagittal planes for the lung and the prostate cases were shown in Figures 5 and 6 with 95%, 80%, 60%, 40%, and 20% isodose distributions represented by the red, yellow, green, light blue, and dark blue lines, respectively. As shown in Figure 5, the 95% red isodose line in EMXRT conformed to the tumor volume (red color organ) better than that in IMRT. In addition, the 40% and 20% isodose lines covered less volume in EMXRT. On the other hand, the IMRT plan delivered a better dose distribution in the prostate case than the EMXRT plan in terms of target dose conformity and normal tissue sparing, as shown in Figure 6.

Tables 5 and 6 compared the dosimetric parameters of PTV and OARs in IMRT plans with those in EMXRT plans for all the six cases. As mentioned early, all plans were normalized such that the 100% isodose lines encompassed 95% of PTV. Overall, the PTV coverage in both EMXRT and IMRT plans showed no big differences, with up to 1% difference in terms of *D*_mean_ and up to 1.7% difference on *D*_2%_ of PTV in the spine case. Compared to IMRT plans, the EMXRT plans showed the same homogeneity of PTV dose distributions indicated by HI values for the prostate, the head and neck, and thyroid cases. For the lung and brain cases, EMXRT plans yielded better PTV dose homogeneity as compared to IMRT, up by 5.9% and 7.1%, respectively. However, the PTV dose homogeneity was 10.5% worse in EMXRT plan of spine lesion when compared with IMRT.

For the prostate case, rectum and bladder received equivalent doses in EMXRT compared with IMRT, in terms of mean dose and *D*_15%_, with up to 3.1% and 1% relative differences, respectively (Table 6). The mean dose to the femur head increased by 29.2% and the *D*_15%_ was up by 42.2% in EMXRT plan, although both indexes were still within the tolerances.

**Figure 7:**
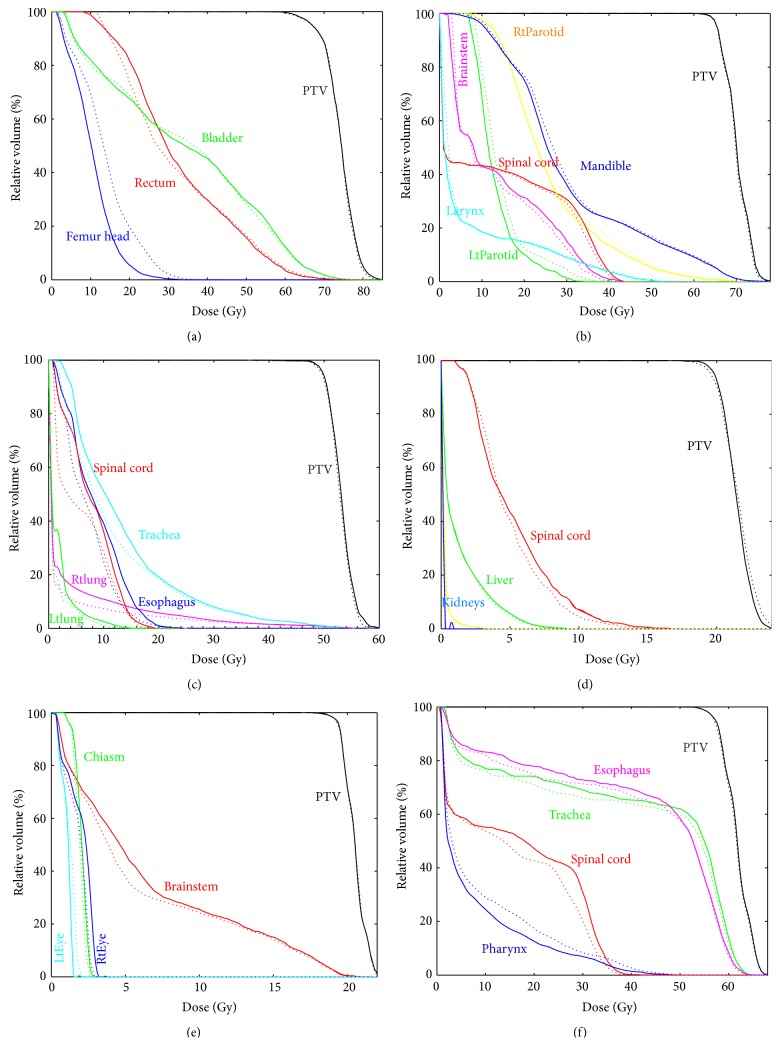
Dose volume histogram (DVH) comparisons between IMRT plans (solid line) and EMXRT plans (dotted line) of (a) prostate cancer, (b) head and neck cancer, (c) lung cancer, (d) spine cancer, (e) brain cancer, and (f) thyroid cancer.

In the head and neck case, all critical structures showed comparable DVHs as indicated in Figure 7(b). Some OARs such as spinal cord, brainstem, and mandible were slightly more spared in EMXRT while, for left and right parotids, the mean doses were 11.2% and 7.1% higher in EMXRT than in IMRT.

In terms of lung cancer radiotherapy, our study indicated that EMXRT was a much better modality than IMRT, not only on the target coverage (Table 5), but also on the OAR sparing (Table 6). The mean dose and maximum dose of the spinal cord were 18.2% and 11.3% lower in EMXRT than in IMRT. The right and left lungs were much more spared with the mean doses decreased from 1.7 Gy and 3.5 Gy to 1.1 Gy and 2.6 Gy, a 35.3% and 25.7% reduction, respectively.

The spinal cord dose in the spine case was a big challenge due to its proximity to the target. In EMXRT plan, with almost the same target coverage, the mean dose to the spinal cord was reduced by 4.1% and the maximum dose got a reduction of 8.2%. Liver in this case also received 7.7% less dose on average as compared to IMRT. However, the target dose coverage was 10.5% less homogeneous in EMXRT plan than in IMRT plan, due to the 5 MV used in the majority of beams for a lesion wrapping around the bony structure.

The brain case in this study was a pediatric case, so any dose reduction on the OARs can be of great significance. Replacing the 6 MV photons in IMRT with the 5 MV photons in EMXRT, it was found that the OARs got better spared yet the PTV dose coverage was 7.1% more homogeneous in EMXRT. Specifically, the mean dose to the brainstem and the maximum doses to the eyes and chiasm were 6.3%, 12.9%, and 7.7% lower in EMXRT than in IMRT. In combination with better target dose coverage, our results suggested that EMXRT with 5 MV photon beams was a better treatment modality for the pediatric brain tumors than the conventional IMRT with 6 MV photon beams.

For the case of thyroid cancer, except for the 21.1% higher mean dose to the pharynx, all the other OARs such as the spinal cord, the esophagus, and the trachea were more spared in EMXRT than in IMRT by 1.7% to 9%. The PTV coverage for the two plans was basically the same.

**Figure 8:**
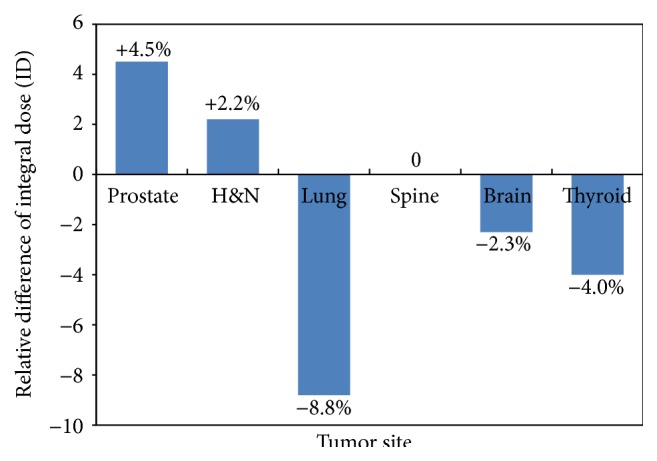
Relative differences of integral dose for the six cases investigated in this study. Positive value means that the EMXRT plan had lower integral dose than that of IMRT plan.

The integral dose defined as the product of absorbed dose with the volume of normal tissues was calculated and compared between the two modalities.  Figure 8 showed the relative difference of integral dose for the six cases. It was obvious that EMXRT outperformed IMRT in the lung, the brain, and the thyroid cases with the reduction of integral dose by 8.8%, 2.3%, and 4.0%, respectively. As to the spine case, both modalities delivered the same amount of integral dose to the total healthy tissues. For the prostate and head and neck cases, IMRT delivered 4.5% and 2.2% less integral doses to the normal tissues than EMXRT did.

## 4. Discussion

In this work, the impact of using mixed lower photon beam for IMRT on six various sites is investigated. Compared to the clinical IMRT energy settings, the proposed mixed lower photon energies plans showed improved target coverage and homogeneity, better sparing of the OARs, and reduced integral dose for the lung cancer and pediatric brain tumor patient. For other lesions such as prostate, spine, thyroid, and head and neck, the lower energy beams were less beneficial than the clinical energy settings.

Beam energies were determined easily by our energy selector according to tumor size and effective path length along beam direction. The lower energy photon beams (<6 MV) help to reduce the doses deposited to the adjacent critical structures in lung cancer EMXRT plan due to the rapid dose fall-off with smaller penumbra at the field edge. The brain case investigated in this work was a pediatric case. Cranial radiation therapy has been associated with the highest risk of long-term cognitive morbidity particularly in younger children. There has been an established dose-response relationship correlating the high OAR dose in cranial radiotherapy with the poor performance on intellectual measures in the long term [23]. The improvements on normal tissue sparing and integral dose reduction are meaningful. Further study will be carried out to explore the role of lower energy photon beams in pediatric cancer radiotherapy.

To verify the primary conclusion of the potential dosimetric benefits of EMXRT for the lung and pediatric brain patients, one more case for each kind of cancer was analyzed and compared. Following the same procedure mentioned above, the energy settings for the EMXRT plans were 4, 5, 5, 3, 3, 4, and 5 MV and 5, 5, 4, 5, 4, 4, and 4 MV for the additional lung and brain cases, respectively, while the corresponding IMRT plans used 6 MV for all beams (Table 7).

**Figure 9:**
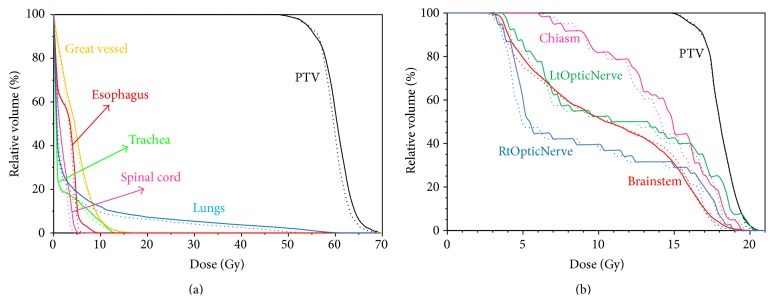
Dose volume histogram (DVH) comparisons between IMRT plans (solid line) and EMXRT plans (dotted line) of (a) the second lung cancer and (b) brain cancer cases.

As shown in Figure 9(a), the EMXRT plan showed better plan quality in the second lung case with steeper PTV dose curve and lower OARs dose curves. The PTV dose was more homogeneous in the EMXRT plan with slightly lower HI value (0.22) compared to the IMRT plan (0.23). The *V*_5 Gy_ of the whole lung tissue, that is, the percent volume of lung receiving 5 Gy, was decreased from 18.8% to 16.9%, with 10.1% reduction in EMXRT compared to that in IMRT, which means more lung tissues were spared by the low dose irradiation. For the EMXRT plan, the mean dose of the spinal cord, esophagus, great vessel, and trachea was decreased by 22.2%, 10.7%, 5.4%, and 4.1%, respectively (Table 8). The integral dose for the second lung case was decreased from 3.02 Gy to 2.73 Gy, with 9.7% reduction for the EMXRT plan. Overall, our conclusion was in agreement with the previous works [8, 9]; that is, the EMXRT and the application of lower energy photons were able to improve the target dose distribution and reduce the OARs dose and the integral dose for the lung cancer treatment.

For the second brain case, the PTV dose distribution was identical for both IMRT and EMXRT with same PTV mean dose and HI value (Figure 9(b) and Table 8). The mean dose to the brainstem was decreased from 9.8 Gy to 9.2 Gy, with a 5.7% reduction in EMXRT compared to that in IMRT. The left and right optic nerves received slightly less doses in EMXRT (2.8% and 2.4% reduction, resp.). The integral dose was 4.6% lower in EMXRT plan (1.44 Gy) than in IMRT (1.51 Gy). Our results suggest that EMXRT has potential dosimetric benefits for the pediatric brain cancer patients.

Although there are currently no technologies available to modulate photon energy, fast energy switching is possible as seen in some of the modern linear accelerators. For example, clinically it only takes less than 10 seconds for the Varian Trilogy to switch energy between 6 MV and 10 MV, making it possible to change energy for different gantry angles during EMXRT treatment. Furthermore, theoretically speaking, the EMXRT modality should provide increased flexibility as photon energy can be treated as a new variable in the planning optimization. Of course, the additional flexibility may increase the difficulty in the optimization and in quality assurance and quality control. Nevertheless, this study has demonstrated the feasibility of EMXRT and clinical benefits of lower energy (<6 MV) photon beams in the lung cancer radiotherapy and pediatric patients, opening a door for a more personalized treatment planning for individual patient.

As mentioned above, there are a growing number of studies on the potential advantages and applications of low energy photon beams (<6 MV) [6, 8, 9]. These energies (<6 MV) were especially considered in this new modality treatment study [24]. With advanced intensity modulation technology, it has been shown that radiotherapy has become less restricted by the weak penetration power of lower energy [25, 26]. The feasibility and potential benefits of EMXRT in the energy range of 2–10 MV have been demonstrated in this study. Higher energy photons (> 10 MV) for EMXRT have not been investigated so far, due primarily to their inherent physical characteristics, such as high exit dose to the critical structures downstream, longer secondary electron range, and neutron contamination.

**Figure 10:**
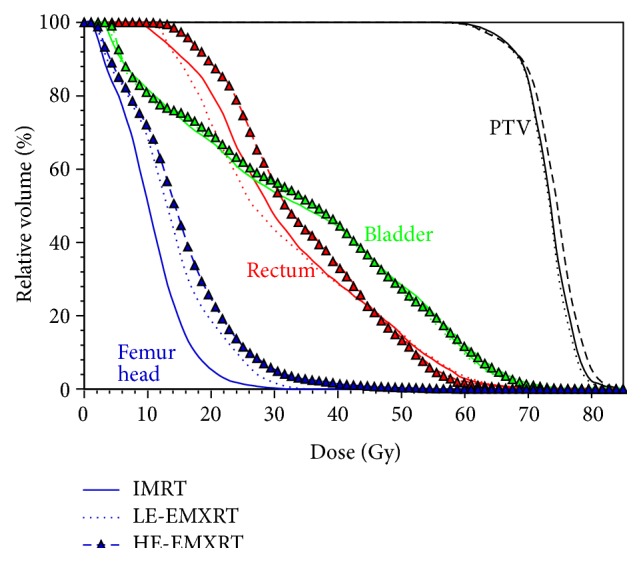
Dose volume histogram (DVH) comparisons between IMRT plan (solid line), LE-EMXRT plan using 5 and 10 MV (dotted line), and HE-EXMRT plan using 5- and 15 MV (dashed line with symbol) of the prostate case.

To study the effect of higher energy photons for EMXRT, we compared a HE-EMXRT (higher energy EMXRT) plan with the LE-EMXRT (lower energy EMXRT) and IMRT plans of prostate cancer. In the LE-EMXRT plan, 10 MV was selected for the left and right posterior oblique beams while 5 MV was used for the anterior posterior and two anterior oblique beams. In the HE-EMXRT plan, the two 10 MV beams were replaced with 15 MV photons. Identical beam configuration and optimization constraints were used in all the three plans. As shown in Figure 10, the target dose distributions were identical for all the plans. However, the rectum received more irradiation in HE-EMXRT plan with mean dose increased by 4.7% compared to that in IMRT plan. The mean dose to the femur head increased by 41.9% in HE-EMXRT plan (13.7 Gy) compared to IMRT plan (10.6 Gy). The increased OAR doses were mostly due to the increased secondary electron range and penetrating power of the 15 MV photons. Our results indicated that, with current energy modulation method, it is not necessary to involve the photon energies higher than 10 MV. While it is possible to further improve the HE-EMXRT plan by adjusting the optimization constraints, we kept them identical in this study for the sake of comparison.

In addition, it has been demonstrated that lower energy mega-voltage photon beams can improve the image quality. The MV fan beam CT (MVCT) with effective energy of 3.5 MV from a Helical Tomotherapy unit has been shown to provide sufficient contrast for soft-tissue delineation [27, 28]. Several studies have further shown that, with low-Z targets in linear accelerators producing photon beams as low as 1.9 MV, the image quality could be greatly enhanced compared to 6 MV photons [29, 30]. Hence, photon beams of lower energy (<6 MV) could be potentially useful for the target localization and radiation delivery in the image-guided IMRT treatments. Furthermore, with the actual treatment beam being the source for MV portal or MVCT image acquisition, the imaging isocenter is identical to the treatment isocenter, eliminating the need to perform a comprehensive calibration and registration procedure usually required if a kilo-voltage imaging system is installed perpendicular to the treatment beam axis [24]. The expanded beam energy range will not only increase the flexibility for personalized radiotherapy, but also provide more options for personalized imaging protocol [31].

## 5. Conclusion

In this study, we have proposed a new treatment modality termed energy modulated photon radiotherapy (EMXRT), which adaptively set the beam energy from a wide range of photon beams (2 to 10 MV) based on patient anatomy and beam angles, and demonstrated its feasibility in six clinical cases by using a Monte Carlo based inverse planning platform. Compared to current IMRT technique, the proposed EMXRT offered much improved OAR sparing in the radiotherapy of lung cancers and pediatric cases. For the prostate, head and neck, spine, and thyroid cases, the EMXRT plans achieved comparable quality to the IMRT plans. Our pilot study suggested that photon beam energy could be added as a new variable in treatment planning for a more personalized radiation treatment and lower energy photons (<6 MV) have the potential benefits in treating lung cancer and pediatric patients. The proposed EMXRT method indicated that photon beam energy may be considered as a technical parameter in current treatment approaches to increase the flexibility of radiotherapy and provide more possibility to perform personalized radiotherapy.

## Figures and Tables

**Table 1:** Characteristics of Monte Carlo simulated percentage depth dose curves for various beam energies. The measured percentage depth dose data were shown in bold.

Relative dose/%	Depth/cm							
	10 MV		6 MV		5 MV	4 MV	3 MV	2 MV
	Monte Carlo	Measured data	Monte Carlo	Measured data	Monte Carlo	Monte Carlo	Monte Carlo	Monte Carlo
100	2.4	**2.4**	1.5	**1.6**	1.3	1.1	0.7	0.5
90	5.6	**5.5**	4.2	**4.2**	3.8	3.3	3.1	2.9
80	8.2	**8.2**	6.6	**6.6**	6.0	5.7	5.2	4.7
70	11.1	**11.1**	9.2	**9.2**	8.6	8.0	7.4	6.9
60	14.5	**14.4**	12.0	**12.0**	11.3	10.4	9.6	9.0
50	18.3	**18.3**	15.0	**15.2**	14.3	13.2	12.2	11.2

**Table 2:** Monte Carlo simulated percentage doses at certain depths for various beam energies. The measured data were listed in the brackets in bold.

Beam index	Photon beam energy					
	10 MV	6 MV	5 MV	4 MV	3 MV	2 MV
% *D*(0)^a^	23.4 **(44.7)**	36.1 **(63.7)**	41.5	51.2	60.5	73.9
% *D*(10)^b^	73.9 **(73.4)**	66.5 **(66.7)**	64.7	61.0	58.7	55.8
% *D*(20)^c^	46.3 **(46.3)**	38.5 **(38.2)**	35.9	32.2	29.1	24.9
% *D*(30)^d^	29.0 **(29.0)**	22.0 **(21.7)**	19.8	16.8	14.3	11.1
TPR_20,10_^e^	0.627 **(0.630)**	0.579 **(0.573)**	0.555	0.528	0.496	0.446

a, b, c, and d are percentage doses at depths of 0, 10, 20, and 30 cm, respectively. e is the tissue phantom ratio calculated as TPR_20,10_ = %  *D*(20)/%  *D*(10).

**Table 3:** Energy table determined by energy selector based on effective path length (EP) and tumor size (TS) in effective diameter. Energies in MV were grouped and illustrated with different superscript letters.

TS/cm	EP/cm																			
	2	3	4	5	6	7	8	9	10	11	12	13	14	15	16	17	18	19	20	21
2	3^a^	3^a^	3^a^	3^a^	3^a^	3^a^	4^b^	4^b^	4^b^	4^b^	4^b^	4^b^	4^b^	4^b^	5^c^	5^c^	10^e^	10^e^	10^e^	10^e^
3		3^a^	3^a^	4^b^	4^b^	4^b^	4^b^	4^b^	4^b^	4^b^	4^b^	4^b^	5^c^	5^c^	5^c^	5^c^	10^e^	10^e^	10^e^	10^e^
4			4^b^	4^b^	4^b^	4^b^	5^c^	5^c^	5^c^	5^c^	5^c^	5^c^	5^c^	5^c^	5^c^	5^c^	10^e^	10^e^	10^e^	10^e^
5			4^b^	5^c^	5^c^	5^c^	5^c^	5^c^	5^c^	5^c^	5^c^	5^c^	5^c^	5^c^	5^c^	5^c^	10^e^	10^e^	10^e^	10^e^
6			5^c^	5^c^	5^c^	5^c^	5^c^	5^c^	5^c^	5^c^	5^c^	5^c^	5^c^	5^c^	5^c^	5^c^	10^e^	10^e^	10^e^	10^e^
7				5^c^	5^c^	5^c^	5^c^	5^c^	5^c^	5^c^	5^c^	5^c^	5^c^	5^c^	5^c^	5^c^	10^e^	10^e^	10^e^	10^e^
8				5^c^	5^c^	5^c^	5^c^	5^c^	5^c^	5^c^	5^c^	6^d^	6^d^	6^d^	6^d^	6^d^	10^e^	10^e^	10^e^	10^e^
9					6^d^	6^d^	6^d^	6^d^	6^d^	6^d^	6^d^	6^d^	6^d^	6^d^	6^d^	6^d^	10^e^	10^e^	10^e^	10^e^
10					6^d^	6^d^	6^d^	6^d^	6^d^	6^d^	6^d^	6^d^	6^d^	6^d^	6^d^	6^d^	10^e^	10^e^	10^e^	10^e^
11						10^e^	10^e^	10^e^	10^e^	10^e^	10^e^	10^e^	10^e^	10^e^	10^e^	10^e^	10^e^	10^e^	10^e^	10^e^
12						10^e^	10^e^	10^e^	10^e^	10^e^	10^e^	10^e^	10^e^	10^e^	10^e^	10^e^	10^e^	10^e^	10^e^	10^e^
13							10^e^	10^e^	10^e^	10^e^	10^e^	10^e^	10^e^	10^e^	10^e^	10^e^	10^e^	10^e^	10^e^	10^e^
14							10^e^	10^e^	10^e^	10^e^	10^e^	10^e^	10^e^	10^e^	10^e^	10^e^	10^e^	10^e^	10^e^	10^e^
15								10^e^	10^e^	10^e^	10^e^	10^e^	10^e^	10^e^	10^e^	10^e^	10^e^	10^e^	10^e^	10^e^
16								10^e^	10^e^	10^e^	10^e^	10^e^	10^e^	10^e^	10^e^	10^e^	10^e^	10^e^	10^e^	10^e^

**Table 4:** Characteristics of the tumors and beam setup of six cases in this study. Beam energies of the original IMRT plans were listed in parallel to those of the EMXRT plans in bold.

Tumor site	*V*_PTV_/cm^3^	*D*_e.s._/cm	*D*_*p*_/Gy	Beam number	Gantry angle	Energy/MV (IMRT)	Energy/MV (EMXRT)	EP/cm	TS/cm
Prostate	119.9	6.1	66.6	1	180	10	**5**	9.9	5.4
				2	225	10	**5**	11.1	6.0
				3	285	10	**10**	20.0	3.8
				4	135	10	**5**	11.3	6.3
				5	75	10	**10**	19.9	6.8

Lung	15.5	3.1	50.0	1	330	6	**4**	10.9	2.2
				2	260	6	**5**	17.2	2.3
				3	240	6	**4**	11.3	2.2
				4	220	6	**4**	8.4	2.2
				5	180	6	**5**	8.7	2.8
				6	250	6	**5**	14.8	2.2
				7	50	6	**5**	15.5	1.7
				8	30	6	**4**	11.6	2.1
				9	10	6	**5**	10.5	2.8

Thyroid	166.6	6.8	60.0	1	250	6	**5**	5.0	7.7
				2	210	6	**4**	3.6	3.5
				3	180	6	**3**	2.5	2.9
				4	150	6	**3**	1.8	2.1
				5	110	6	**5**	5.3	7.7

Brain	50.2	4.6	20.0	1	310	6	**5**	7.5	5.3
				2	110	6	**5**	6.8	5.4
				3	75	6	**5**	6.6	5.5
				4	50	6	**5**	6.4	5.0
				5	0	6	**5**	6.2	4.7

Spine	17.5	3.2	20.0	1	345	6	**5**	7.3	3.8
				2	320	6	**5**	8.8	4.1
				3	290	6	**5**	15.3	2.7
				4	235	6	**5**	15.3	4.1
				5	210	6	**5**	15.5	4.1
				6	180	6	**5**	16.6	4.0
				7	160	6	**10**	17.8	4.0
				8	130	6	**5**	17.4	4.2
				9	70	6	**5**	12.8	3.9
				10	40	6	**5**	9.7	4.0
				11	15	6	**5**	8.0	3.9

Head & neck	193.0	7.2	68.0	1	340	6	**4**	8.5	2.2
				2	310	6	**5**	8.4	3.1
				3	255	6	**5**	5.7	4.4
				4	215	6	**4**	7.0	2.9
				5	180	6	**4**	8.2	2.3
				6	145	6	**4**	7.1	2.8
				7	105	6	**5**	4.7	6.1
				8	50	6	**4**	7.1	3.1
				9	20	6	**5**	8.3	2.8

*V*_PTV_ is the volume of PTV.  *D*_e.s._ is the diameter of equivalent sphere of PTV volume.  *D*_*p*_ is the prescription dose for PTV.  EP is effective path length.  TS is tumor size.

**Table 5:** Comparison of PTV indices between IMRT plan and EMXRT plan for the six cases in this study.

Tumor site	Indices	IMRT/Gy	EMXRT/Gy	Relative difference^*∗*^/%
Prostate	*D*_95%_	66.6	66.6	0.0
	*D*_mean_	74.5	74.1	−0.5
	*D*_98%_	63.9	62.9	−1.6
	*D*_2%_	80.1	79.0	−1.4
	HI	0.22	0.22	0.0

Head and neck	*D*_95%_	68	68.0	0.0
	*D*_mean_	72.1	71.9	−0.3
	*D*_98%_	67.8	67.8	0.0
	*D*_2%_	79.2	79.4	+0.3
	HI	0.16	0.16	0.0

Lung	*D*_95%_	50.0	50.0	0.0
	*D*_mean_	53	52.9	−0.2
	*D*_98%_	48.8	48.5	−0.6
	*D*_2%_	57.6	56.9	−1.2
	HI	0.17	0.16	−5.9

Spine	*D*_95%_	20.0	20.0	0.0
	*D*_mean_	20.6	20.8	+1.0
	*D*_98%_	19.4	19.4	0.0
	*D*_2%_	23.5	23.9	+1.7
	HI	0.19	0.21	+10.5

Brain	*D*_95%_	20.0	20.0	0.0
	*D*_mean_	20.5	20.5	0.0
	*D*_98%_	19.6	19.8	+1.0
	*D*_2%_	22.5	22.6	+0.4
	HI	0.14	0.13	−7.1

Thyroid	*D*_95%_	68.0	68.0	0.0
	*D*_mean_	72.1	71.9	−0.3
	*D*_98%_	67.8	67.8	0.0
	*D*_2%_	79.2	79.4	0.3
	HI	0.16	0.16	0.0

^*∗*^Relative difference = 100%  ×  (Index_EMXRT_  −  Index_IMRT_)/Index_IMRT_. *D*_mean_, *D*_max_, and *D*_min_ are the mean, maximum, and minimum doses of PTV, respectively. *D*_98%_ and *D*_2%_ are the dose levels covering 98% and 2% of the PTV volume, respectively. HI = (*D*_2%_ − *D*_98%_)/*D*_50%_.

**Table 6:** Comparison of dose indices of the organs-at-risk (OARs) between the IMRT and the EMXRT plans for the six cases in this study.

Tumor site	OARs	Indices	IMRT/Gy	EMXRT/Gy	Relative difference^*∗*^/%
Prostate	Rectum	*D*_mean_	32.5	31.5	−3.1
		*D*_15%_	49.7	50.7	+2.1
	Bladder	*D*_mean_	34.0	33.8	−0.6
		*D*_15%_	57.9	57.3	−1.0
	Femur head	*D*_mean_	10.6	13.7	+29.2
		*D*_15%_	15.4	21.9	+42.2

Head and neck	Spinal cord	*D*_mean_	14.3	13.9	−2.8
		*D*_max_	44.7	45.2	+1.1
	Brainstem	*D*_mean_	14.0	13.5	−3.6
		*D*_max_	44.6	43.1	−3.4
	Mandible	*D*_mean_	31.2	31.6	+1.3
		*D*_max_	82.1	80.3	−2.2
	Left parotid	*D*_mean_	13.4	14.9	+11.2
	Right parotid	*D*_mean_	26.7	28.6	+7.1

Lung	Spinal cord	*D*_mean_	7.7	6.3	−18.2
		*D*_max_	19.4	17.2	−11.3
	Left lung	*D*_mean_	3.5	2.6	−25.7
	Right lung	*D*_mean_	1.7	1.1	−35.3
	Trachea	*D*_mean_	13.1	12.9	−1.5
	Esophagus	*D*_mean_	8.5	7.4	−12.9

Spine	Spinal cord	*D*_mean_	4.9	4.7	−4.1
		*D*_max_	15.9	14.6	−8.2
	Liver	*D*_mean_	1.3	1.2	−7.7
	Kidneys	*D*_mean_	0.3	0.3	0.0

Brain	Brainstem	*D*_mean_	6.3	5.9	−6.3
		*D*_max_	20.2	20.2	0.0
	Eyes	*D*_max_	3.1	2.7	−12.9
	Chiasm	*D*_max_	2.6	2.4	−7.7

Thyroid	Spinal cord	*D*_mean_	17.6	16.0	−9.0
		*D*_max_	41.6	40.6	−2.3
	Esophagus	*D*_mean_	44	43.3	−1.7
	Trachea	*D*_mean_	43.2	42.0	−2.7
	Pharynx	*D*_mean_	8	9.7	+21.1

^*∗*^Relative difference = 100%  ×  (Index_EMXRT_  −  Index_IMRT_)/Index_IMRT_. The “+” and “−” indicate whether the dose index of the EMXRT plan is larger or smaller than that of IMRT plan. *D*_mean_ is the mean dose, and *D*_max_ is the maximum dose.

**Table 7:** Characteristics of the beam setup of the extra lung and brain case.

Patients	Lung	Brain
# of beams	7	7
EP/cm	7.1, 7.0, 8.0, 4.0, 4.1, 6.8, and 8.5	8.0, 8.1, 7.4, 8.0, 7.1, 7.2, and 7.2
TS/cm	4.2, 5.1, 4.5, 3.2, 3.2, 3.8, and 4.5	4.0, 4.1, 3.0, 4.1, 3.2, 3.6, and 3.7
Energy/MV (IMRT)	6, 6, 6, 6, 6, 6, and 6	6, 6, 6, 6, 6, 6, and 6
Energy/MV (EMXRT)	**4**, **5**, **5**, **3**, **3**, **4**, and **5**	**5**, **5**, **4**, **5**, **4**, **4**, and **4**

EP is effective path length. TS is tumor size.

**Table 8:** Comparison of dosimetric indices between the IMRT and the EMXRT plans for the extra lung and brain case.

Case	Tumor site	Indices	IMRT	EMXRT	Relative difference^*∗*^/%
Extra lung	PTV	*D*_95%_/Gy	54.0	54.0	0.0
		*D*_mean_/Gy	54.5	54.1	−0.7
		HI	0.23	0.22	−6.6
	Trachea	*D*_mean_/Gy	1.71	1.64	−4.1
	Great vessel	*D*_mean_/Gy	4.09	3.87	−5.4
	Spinal cord	*D*_mean_/Gy	1.53	1.19	−22.2
	Esophagus	*D*_mean_/Gy	2.62	2.34	−10.7
	Lungs	*D*_mean_/Gy	4.39	3.77	−14.1
		*V*_5 Gy_	18.80%	16.90%	−10.1
		*V*_20 Gy_	6.92%	6.70%	−0.3

Extra brain	PTV	*D*_95%_/Gy	16.1	16.1	0.0
		*D*_mean_/Gy	16.0	16.0	0.0
		HI	0.23	0.23	0.0
	Chiasm	*D*_mean_/Gy	12.6	12.5	−0.8
	Brainstem	*D*_mean_/Gy	9.8	9.2	−5.7
	LtOpticNerve	*D*_mean_/Gy	10.7	10.4	−2.8
	RtOpticNerve	*D*_mean_/Gy	8.3	8.1	−2.4

^*∗*^Relative difference = 100%  ×  (Index_EMXRT_ − Index_IMRT_)/Index_IMRT_. *D*_mean_ is the mean doses of each organ/tissue. HI = (*D*_2%_ − *D*_98%_)/*D*_50%_. *D*_98%_, *D*_2%_, and *D*_50%_ are the dose levels covering 98%, 2%, and 50% of the PTV volume, respectively. *V*_5 Gy_ and *V*_20 Gy_ are the percent of whole lung volume receiving 5 Gy and 20 Gy dose, respectively.
